# Volatile Profiling of Spirulina Food Supplements

**DOI:** 10.3390/foods13081257

**Published:** 2024-04-19

**Authors:** Aikaterina Paraskevopoulou, Triantafyllos Kaloudis, Anastasia Hiskia, Martin Steinhaus, Dimitra Dimotikali, Theodoros M. Triantis

**Affiliations:** 1 Institute of Nanoscience and Nanotechnology, National Center for Scientific Research “Demokritos”, Patriarchou Grigoriou E & 27 Neapoleos Str., Agia Paraskevi, 15341 Athens, Greece; k.paraskevopoulou@inn.demokritos.gr (A.P.); t.kaloudis@inn.demokritos.gr (T.K.); a.hiskia@inn.demokritos.gr (A.H.); 2 School of Chemical Engineering, National Technical University of Athens, Iroon Politechniou 9, Zografou, 15780 Athens, Greece; demot@chemeng.ntua.gr; 3 Department of Water Quality Control, Athens Water Supply and Sewerage Company (EYDAP SA), 156 Oropou Str., 11146 Athens, Greece; 4 Leibniz Institute for Food Systems Biology at the Technical University of Munich (Leibniz-LSB@TUM), Lise-Meitner-Straße 34, 85354 Freising, Germany; martin.steinhaus@tum.de

**Keywords:** volatile organic compounds (VOCs), spirulina biomass, spirulina food supplements, headspace solid-phase microextraction (HS-SPME), gas chromatography-mass spectrometry (GC-MS), untargeted analysis

## Abstract

Spirulina, a cyanobacterium widely used as a food supplement due to its high nutrient value, contains volatile organic compounds (VOCs). It is crucial to assess the presence of VOCs in commercial spirulina products, as they could influence sensory quality, various processes, and technological aspects. In this study, the volatile profiles of seventeen commercial spirulina food supplements were determined using headspace solid-phase microextraction (HS-SPME), coupled with gas chromatography-mass spectrometry (GC-MS). The identification of volatile compounds was achieved using a workflow that combined data processing with software tools and reference databases, as well as retention indices (RI) and elution order data. A total of 128 VOCs were identified as belonging to chemical groups of alkanes (47.2%), ketones (25.7%), aldehydes (10.9%), alcohols (8.4%), furans (3.7%), alkenes (1.8%), esters (1.1%), pyrazines (0.8%), and other compounds (0.4%). Major volatiles among all samples were hydrocarbons, especially heptadecane and heptadec-8-ene, followed by ketones (i.e., 4-(2,6,6-trimethyl-1-cyclohexen-1-yl)-3-buten-2-one, β-ionone, 2,2,6-trimethylcyclohexan-1-one), aldehydes (i.e., hexanal), and the alcohol oct-1-en-3-ol. Several volatiles were found in spirulina dietary supplements for the first time, including 6,10-dimethylundeca-5,9-dien-2-one (geranylacetone), 6,10,14-trimethylpentadecan-2-one, hept-2-enal, octanal, nonanal, oct-2-en-1-ol, heptan-1-ol, nonan-1-ol, tetradec-9-en-1-ol, 4,4-dimethylcyclohex-2-en-1-ol, 2,6-diethylpyrazine, and 1-(2,5-dimethylfuran-3-yl) ethanone. The methodology used for VOC analysis ensured high accuracy, reliability, and confidence in compound identification. Results reveal a wide variety of volatiles in commercial spirulina products, with numerous newly discovered compounds, prompting further research on sensory quality and production methods.

## 1. Introduction

In recent decades, the global market has witnessed a shift in consumer preferences towards healthier lifestyles. This shift has led to a growing interest in producing natural food products devoid of chemical preservatives and other additives. One highly sought-after natural ingredient in the food industry is spirulina, a cyanobacterium belonging to the genus *Arthrospira* [1]. Spirulina is prized for its rich nutrient profile, encompassing proteins, vitamins, pigments, and favorable fatty acids [2,3,4]. Beyond its primary use as a dietary supplement, spirulina also finds application as a natural additive in various food products, enhancing their nutritional value [5,6,7]. Moreover, the versatility of spirulina extends into the pharmaceutical and cosmetic industries [8,9], where it is valued for its antimicrobial [10], anticancer [11], antibacterial, and antioxidant properties [4,12]. Despite its impressive nutritional profile, consumer acceptance of spirulina may still face challenges due to its undesirable fishy odor [13]. This characteristic smell has been associated mainly with a mixture of volatile organic compounds (VOCs) such as 6-, 8- and 9-carbon aldehydes, ketones, and alcohols [13,14,15,16].

VOCs are secondary metabolites produced by cyanobacteria, including spirulina. Due to their low molecular weight and high vapor pressure, they are easily released into the atmosphere [15,17,18]. Their synthesis involves several biosynthesis pathways, including the action of enzymes or degradation reactions [19]. Spirulina can grow in photoautotrophic (in the light), heterotrophic (on an organic carbon source), and mixotrophic (simultaneously in the light and on an organic carbon source) cultures [20,21]. Depending on the cultivation mode, the VOC content of a produced biomass is affected by the availability of carbon sources (inorganic/organic), nitrogen, and phosphorus, as well as the light photon flux, temperature, and pH conditions [15]. Limited studies on the volatile compounds associated with raw spirulina biomass are currently available. Based on these studies, VOCs are most frequently detected in chemical groups including saturated hydrocarbons, particularly medium-chain alkanes (C_14_–C_17_), along with alcohols, ketones, and esters [22,23,24,25,26]. However, when it comes to understanding the volatile composition of spirulina food supplements, the available data are quite restricted, primarily originating from specific regions [17,27,28]. The identified VOCs in these studies align with the aforementioned chemical groups, producing evidence of the presence of aldehydes, pyrazines, furans, and furanones, which are also part of the volatile profile of commercial spirulina. Previous studies have tried to link the wide range of volatiles with the unpleasant odor of spirulina [17,22,26]. The presence of compounds like hexadecanamide and palmitic acid derivatives [13] or oct-1-en-3-ol [22] in spirulina biomass was associated with its fishy smell or off-odor [13,29], although there is insufficient data to firmly support these claims. In general, the majority of volatiles present in a food product do not contribute to its smell. In most cases, the overall odor derives not from a single compound, but from a mixture of different odorants [30].

Studies on volatile organic compounds in spirulina have employed different extraction methods, such as hydrodistillation [24,25,27], Soxhlet apparatus extraction [28], supercritical carbon dioxide extraction [31] and headspace solid-phase microextraction (HS-SPME) [13,17,22,23,26]. Notably, HS-SPME stands out among these techniques as it allows a one-step process, enabling the automation and streamlining of the extraction procedure in terms of time and cost-effectiveness [13,17,22,23]. The analysis of VOCs in spirulina by gas chromatography coupled with mass spectrometry (GC-MS) poses considerable difficulties due to the broad spectrum of structurally diverse compounds, frequently found at deficient concentrations. In many cases, the identification of VOCs lacks the integration of mass spectra with data from spectral deconvolution, retention indices (RI), and retention time data from different columns [13,22]. This absence of combined methodologies leads to a low confidence level in volatile identification and increases the risk of misidentifications. Furthermore, recent data have indicated that numerous VOCs may possess various biological activities, including antimicrobial, anticancer, and antioxidant properties, offering potential health benefits, and opening up new opportunities for applications beyond their conventional role as food additives [11,24,25,32]. Therefore, there is a pressing need for a comprehensive analysis and the development of a suitable approach to accurately characterize the VOC profile in spirulina food supplements.

This study aimed to comprehensively elucidate the volatile composition of various commercial spirulina food supplements obtained from different sources. To achieve this, we utilized HS-SPME-GC-MS analysis, coupled with data processing, through the NIST database, retention indices and software tools, ensuring enhanced accuracy, reliability, and confidence in identifying VOCs. Our objective was to extend the current knowledge of the presence of VOCs in spirulina products, thereby enabling further improvements in production processes, particularly concerning sensory quality and additional beneficial properties, to enhance their market positioning.

## 2. Materials and Methods

### 2.1. Spirulina Samples, Reference Substances, and Solvents

The analyzed samples (17 in total) included sixteen commercial spirulina products from various producers and geographical origins, collected from stores in Athens, Greece and one raw biomass sample (Table 1, sample S2), obtained from a local Greek producer. Analytical standards of 4-(2,6,6-trimethyl-1-cyclohexen-1-yl)-3-buten-2-one (*β*-ionone), pentan-1-ol, hexanal, hexan-1-ol, heptan-2-one, benzaldehyde, butan-1-ol, 1,3,3-trimethyl-2-oxabicyclo[2.2.2]octane (eucalyptol), 2,4-di-*tert*-butylphenol, butan-2-one, naphthalene, 1-isopropyl-4-methyl-1,4-cyclohexadiene (*γ*-terpinene), heptan-1-ol, oct-1-en-3-ol, 2,6,6-trimethyl-1-cyclohexene-1-carboxaldehyde (*β*-cyclocitral), octan-1-ol, tetradecane, 4-(2,6,6-trimethylcyclohex-2-en-1-yl)but-3-en-2-one (*α*-ionone), pentadecane, pent-1-en-3-one, hexadecane, and heptadecane, were obtained from Sigma-Aldrich; Merck KGaA (Darmstadt, Germany). A standard mixture of these twenty-two compounds was prepared at a concentration of 10 μg/L in ultrapure water. A standard mixture of alkanes (C7–C30) from Supelco; Merck KGaA (Darmstadt, Germany) was used for retention index calibration. Ultrapure water (18.2 MΩ) was obtained from a Purelab Ultra system (Elga - LabWater, High Wycombe, UK).

### 2.2. Sample Preparation

Powder and flake samples were mixed thoroughly in their containers before sampling. From each container, 5 tablets/capsules or 1 g of flakes/powder were sampled and pulverized to a fine powder using a Yellowline A10 analytical grinder universal mill (IKA, Wilmington, NC, USA).

### 2.3. HS-SPME-GC/MS Analysis

The extraction of volatile compounds was carried out by headspace solid-phase microextraction (HS-SPME), using a PAL-RSI autosampler (PALsystem, CTC Analytics, Zwingen, Switzerland). VOCs were analyzed using a 456 Scion GC, coupled with a TQ mass spectrometer (Bruker Daltonics, Bremen, Germany). In total, 50 mg of the sample was transferred into a 20 mL SPME glass vial (Wheaton MicroLiter) containing 3 g NaCl, followed by the addition of 10 mL of ultrapure water. Blanks were prepared in the same way without adding a sample. The vials were tightly sealed with screw top caps with PTFE/silicon septa. HS-SPME was performed automatically, according to the following conditions: (a) pre-incubation for 10 min at 60 °C; (b) headspace extraction by a 2 cm Divinylbenzene/Carboxen/Polydimethylsiloxane SPME fiber (DVB/CAR/PDMS-50/30 μm) (Supelco; Merck KGaA, Darmstadt, Germany), for 10 min at 60 °C; (c) desorption by exposing the fiber into the GC injector for 2 min at 250 °C. All samples were injected in splitless mode. Helium was used as the carrier gas, with a constant flow of 1.0 mL/min. GC analysis was carried out using two types of columns, a non-polar column RXI^®^—5 Sil MS—(30 m, 0.25 mm ID, 0.25 μm film thickness; Restek, Bellefonte, PA, USA) (column A) and a semi-polar column RXI^®^—624 Sil MS—(60 m, 0.32 mm ID, 1.8 μm film thickness, Restek) (column B). Two different temperature gradients were used. The temperature for column A was initially 50 °C for 1 min, then was raised to 250 °C (15 °C/min), and maintained at 250 °C for 5 min. The total run time was 19.33 min. For column B, the initial temperature was 50 °C for 5 min, then raised to 250 °C (8 °C/min), and maintained for 10 min (total run time 40 min). The mass spectrometry conditions were electron ionization (EI) at 70 eV, positive polarity, full-scan mode, and *m*/*z* scan range 30–300 for column A and 30–400 for column B.

### 2.4. Data Analysis

Mass spectrometry data processing was performed using MSWS 8 software (Bruker). The identification of VOCs was carried out with AMDIS ver. 2.73 (NIST) and the NIST mass spectral library and Search Software, ver. 2017. AMDIS was used for mass spectral deconvolution and the correction of the match factor NET with retention index data from a target NIST library (RI(lib)). AMDIS calculated the difference (RI–RI(lib)), where RI represents the experimental retention index for each unknown compound. This index was calculated based on the calibration of the chromatographic system by analyzing a mixture of C7–C30 saturated alkanes under the same chromatographic conditions as samples. This is a standard procedure in untargeted GC-MS analysis, aiming to express the retention data in a standardized system, independent of the instrument parameters. The retention times of alkanes eluted before and after an unknown compound were used to calculate its experimental RI value based on the equation proposed by H. van den Dool and D.J. Kratz [33]. When the difference (RI–RI(lib)) was higher than a default value (set at 20) [34], then a penalty was applied to the NET value. The criterion for achieving a certain level of identification (Table 2) was set to NET > 80 [34,35]. A lower confidence of identification (annotation) was achieved when using column B, since no RI data were available for this column. In this case, annotation was considered when NET was >80. Using all available data, six levels of identification were defined, as shown in Table 2.

Samples were analyzed in duplicate, and the peak areas were averaged. Normalization was employed to express the relative abundance (%) (arbitrary area units) of individual compounds or chemical groups within each sample, relative to the total peak area of the identified VOCs in that sample (column A). The average relative abundance (%) of each chemical group among all samples using the same column was calculated relative to the average peak area of all identified VOCs in each sample [15,27].

## 3. Results and Discussion

### 3.1. Identification of VOCs

**Scheme 1:**
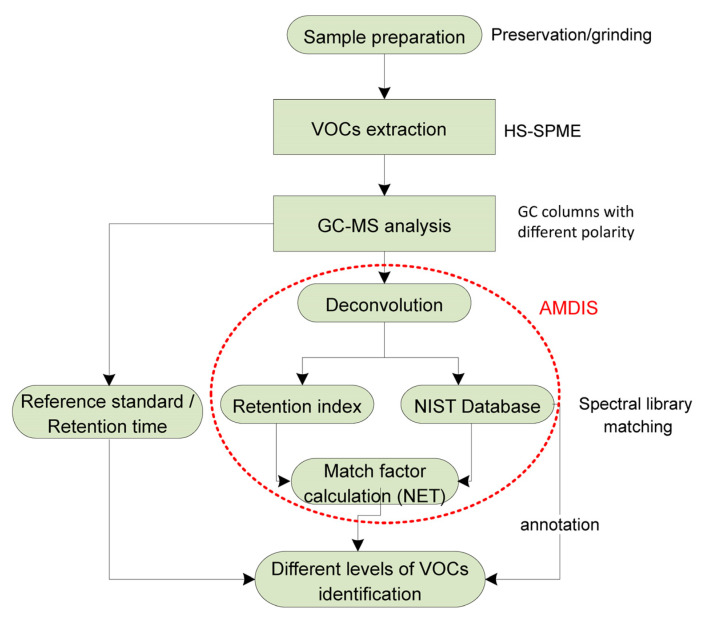
Workflow for the analysis of VOCs in spirulina food supplements.

We developed a comprehensive workflow to analyze and identify volatile compounds in spirulina food supplements (Scheme 1). The process commenced with sample preparation, involving homogenizing and grinding the biomass. Subsequently, automated HS-SPME was used for the volatile compound extraction and GC-MS analysis was carried out, with the resulting data being processed using AMDIS software ver. 2.73. AMDIS served the purpose of deconvoluting the MS spectra, determining the experimental Retention Index (RI) of each deconvoluted component (when RI(lib) was available) and matching the deconvoluted mass spectra against the NIST library.

The combination of mass spectral and retention data allowed us to calculate a match factor, called NET. Achieving a NET value close to 100 resulted in a higher match quality and more reliable identifications. Conversely, NET values significantly decreased when the experimental RI differed significantly from the RI(lib), effectively minimizing false-positive results by filtering out candidate compounds when NET values were below 80. Furthermore, integrating RI data with spectral matching facilitated the identification of isobaric compounds, a challenging task when relying solely on mass spectral data [35].

In cases where RI data were unavailable, we relied solely on mass spectral matching for component annotation. We further employed a second column with different polarities to confirm results and annotate substances that the first column may have missed. However, for the complete identification of a suspected compound (Levels A, B in Table 2), we compared its retention time (t_R_) with that of an authentic standard analyzed under the same conditions (±0.10 min), in addition to mass spectral matching.

**Figure 1:**
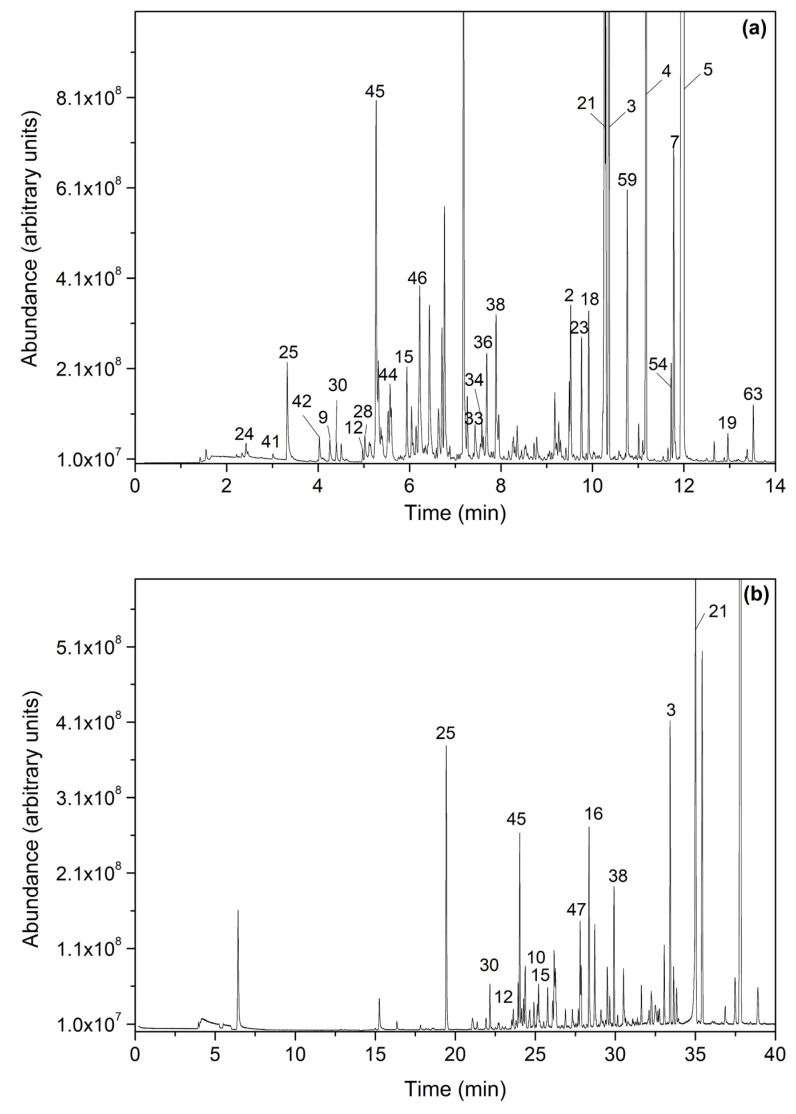
GC-MS total ion chromatograms of spirulina sample S10 extract after deconvolution with AMDIS using (**a**) column A and (**b**) column B. The numbered peaks correspond to the compound numbers listed in the first column of Table 3.

Figure 1 shows representative total ion GC-MS chromatograms (Total Ion Current, TIC) obtained from spirulina sample S10 extract (Table 1), after processing with AMDIS using different columns. It is the first time that two analytical columns of different polarities were used to determine spirulina volatiles. After analysis of the sample extract using the non-polar GC column A, 51 compounds were identified as shown in Figure 1a. Six compounds were found only with the semi-polar GC column B, while twenty compounds were identified with both columns (Figure 1b). The use of a second column with dissimilar polarity for the confirmation of the identified compounds provided further confidence in identification. This technique produces a lower rate of false positive results but does not eliminate them [36].

**Figure 2:**
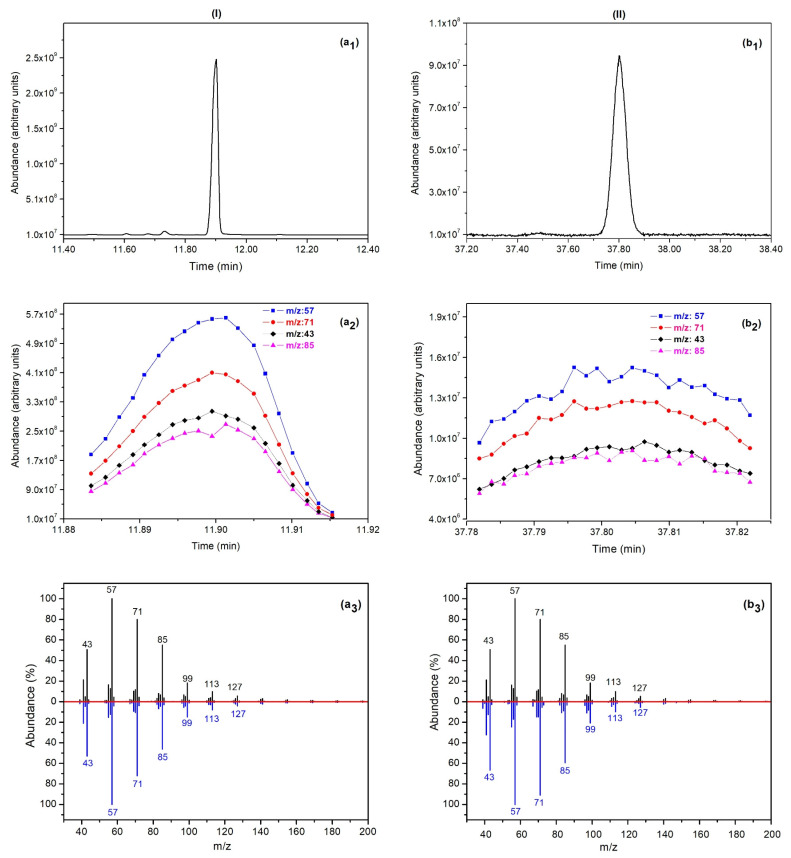
Details of the AMDIS analysis for identification of heptadecane in sample S1 using column A (I) and column B (II); (**a_1_**,**b_1_**) deconvoluted total ion chromatograms; (**a_2_**,**b_2_**) major characteristic ions; (**a_3_**,**b_3_**) spectral matching between the deconvoluted spectrum (blue lines) and the spectrum from the NIST library (black lines).

The identification of heptadecane in sample S1 is illustrated in Figure 2 as an example. Heptadecane was identified (Level A, Table 2) in all spirulina supplements. In all cases, a component peak was obtained (Figure 2a_1_,b_1_) based on the major characteristic ions (*m*/*z*: 43, 57, 71, 85) of the deconvoluted spectra (Figure 2a_2_,b_2_). In the case of column A, spectral matching using the NIST library (Figure 2a_3_), in combination with retention index scoring (RI-RI(lib)), resulted in the highest match factor (NET 100). The NET value was lower (87) when using column B (Figure 2b_3_), reflecting the lower matching of mass spectra, due to higher baseline signals (column bleed). Although higher match factors were generally obtained with column A, further confirmation with column B reduced the risk of false positives.

### 3.2. VOCs in Commercial Spirulina Samples

The screening of spirulina supplements revealed a wide variety of compounds (128 in total). In particular, 68 volatiles were found in four or more samples, while the remaining compounds were found in one to three spirulina supplements. The detailed list of these frequently detected 68 VOCs is presented in Table 3. The volatile profiles of each sample, including the total number of detected compounds, are shown in detail in Table S1 (column A) and Table S2 (column B). The chemical classes of these VOCs included alkanes, alkenes, ketones, aldehydes, alcohols, pyrazines, furans/furanones, esters, and others, demonstrating the high diversity of VOCs present in commercial spirulina products.

To the best of our knowledge, this study uncovered several compounds from various chemical classes that have not been previously reported in commercial spirulina products. Our workflow facilitated the identification of isobaric compounds using RI filtering through the combination of retention data and mass spectra. For example, this approach identified 3,4-dimethylbenzaldehyde and 3-ethylbenzaldehyde (Table 3), which had not been reported before. Octan-2-ol (Table S1) was also identified, for the first time, in commercial spirulina products and successfully differentiated from octan-1-ol (Table 3) using RI data, elution order information, and mass spectra. Similarly, *α*- and *β*-cyclocitral were distinguished by their different RI values. The presence of isobaric compounds with nearly identical mass spectra, as seen in the case of *α*-ionone and *γ*-ionone, added complexity to the identification process. In these cases, using retention data substantially increased the identification confidence [39].

**Figure 3:**
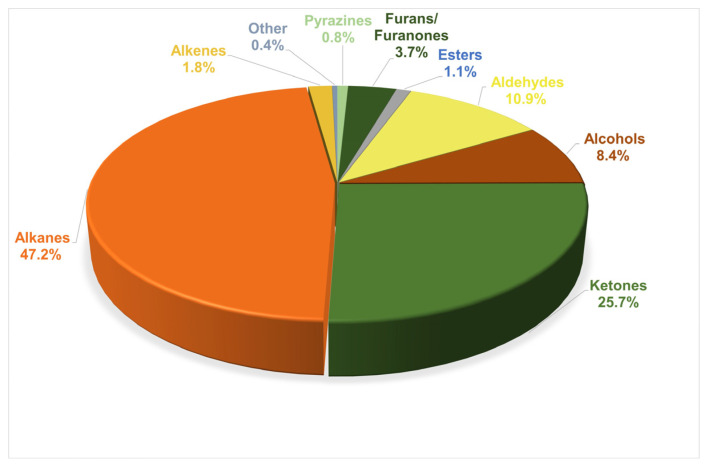
Chemical classes of VOCs and their average relative abundances (%) among all commercial spirulina supplements (column A).

Alkanes were the main chemical group, with an average relative abundance of 47.2%. The relative composition of VOCs was calculated considering both the abundance of each compound of a chemical class and the frequency of occurrence in the samples analyzed (Figure 3). Among all samples, the average relative abundance of ketones, aldehydes, alcohols, furans/furanones, and alkenes was 25.7, 10.9, 8.4, 3.7, and 1.8%, respectively. Significantly lower percentages were calculated for esters, pyrazines, and other compounds (≤1.1%) (Figure 3).

These results align with data from earlier studies, which also identified the presence of similar chemical classes of VOCs in spirulina samples. In most instances, the predominant class observed was that of alkanes [17,22,25,26]. The highest number of VOCs (76) in commercial spirulina products was reported in the study of Aguero et al. [27], followed by Ramasamy et al. [28] who detected seventy-four different compounds in polar and non-polar solvent extracts of a dried spirulina supplement. Martelli [17] identified sixty-one different VOCs in a commercial *Arthrospira platensis* sample. The highest number of volatiles in raw spirulina biomass was ninety-five [26], while in other cases, the number of identified VOCs was lower than seventy [25].

**Figure 4:**
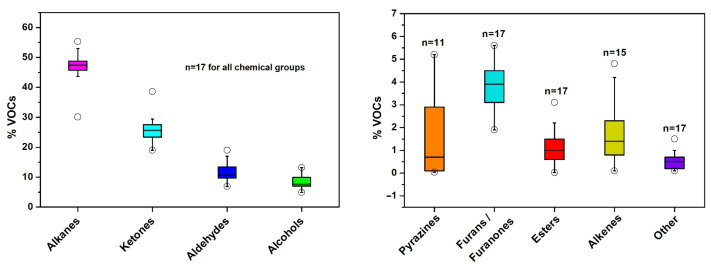
Boxplots (quartiles, median) showing the distribution of VOC groups among samples (column A). The *n* indicates the number of samples in which compounds of a chemical group were detected.

The abundance of the various VOC chemical groups in spirulina supplements is shown in Figure 4. Alkanes varied from 30.1 to 55.3%, with a median of 47.4%. Results are in good agreement with a previous study where hydrocarbons were the main group in *Arthrospira platensis*, with an abundance of 60.7% [26]. Hydrocarbons also constituted the primary chemical group in numerous other studies on spirulina, with percentages ranging from 37.6% [27] to 72.8% [15]. Differences within the same chemical class across various studies can be attributed to variations in growth conditions, different *Arthrospira* species, and variations in the analytical workflows employed [13,15]. Ketones emerged as the second most abundant chemical class, with relative abundances ranging from 19.0 to 38.6% (median 25.6%) as depicted in Figure 4. Consistent with these findings, other studies also confirmed the presence of ketones (including ionones), in spirulina samples, ranking as the second most abundant group following hydrocarbons, with abundances ranging from 14.1 to 22.1% [15,27]. Additionally, our study identified aldehydes (6.9 to 19.0%; median 10.7%) and alcohols (4.9 to 12.6%; median 7.7%) as distinct groups (Figure 4). These results align with a prior study, which reported abundances of 5.5% for aldehydes and 8.6% for alcohols [26].

Chemical groups found at low relative abundances exhibited more significant variation across samples, as shown in Figure 4. For instance, pyrazines were detected in only eleven samples, with a median abundance of 0.7%, consistent with findings from previous studies [27]. However, this study identified a higher number of individual pyrazine derivatives. Furans/furanones, on the other hand, were identified in all spirulina supplements, with abundances ranging from 1.9 to 5.6% (median 3.9%) (Figure 4), comparable to previous reports [26]. Esters were identified with a mean relative area, ranging from 0.02 to 2.5% (median 1%), comparable to previous reports [15,26]). Notably, our study identified a greater number of esters (10 in total, detailed in Table 3 and Table S1) and it appears that the presence of each compound was sample-specific. A limited number of alkenes were identified in fifteen samples (abundances of 0.4 to 4.8%) with heptadec-8-ene making the most significant contribution, as reported previously [15]. Other compounds mainly included pyridine derivatives, exhibiting very low abundances in most samples (Figure 4).

Only a limited subset of VOCs was found in all samples, specifically, 16 out of the total 128 (indicated with a number sign in Tables S1 and S2). This finding suggests that the VOC profiles of spirulina may vary, depending on the species and environmental conditions under which it was cultivated, given the diverse geographical origins of the samples [13,40]. The processing of food supplements, especially through thermal processes used for drying biomass, can trigger various chemical reactions such as Maillard reactions, Strecker degradation, and oxidation, whether mediated by enzymes or not. Additionally, processing dry biomass in an oxygen-rich environment may induce alterations in the final product [41]. In summary, the primary VOC chemical groups observed in spirulina samples were alkanes, ketones, aldehydes, and alcohols, consistent with previous studies [13,15,26]. These chemical classes include numerous compounds which are considered significant contributors to the sensory properties of spirulina biomass and are tentatively associated with its flavor (Table 3) [13]. In the subsequent sections, a comprehensive discussion will be provided regarding specific VOCs in spirulina food supplements, along with an exploration of their potential sources.

**Alkanes–Alkenes**: This study detected twenty-two hydrocarbons, with pentadecane, hexadecane, and heptadecane occurring in all samples. Heptadecane was the most abundant hydrocarbon, with relative abundances ranging from 10.5 to 48.7%. Pentadecane and hexadecane exhibited lower abundancies (1.3–17.0% and 0.4–18.6%, respectively). Tetradecane and tridecane were present in ten and four samples, respectively (Table 3). Another important hydrocarbon, previously identified in cyanobacterial strains *Nostoc* and *Anabaena* [23], and found in very low levels in spirulina essential oil [25], was heptadec-8-ene, detected in fourteen samples in our study. Other alkenes, including the monoterpenes limonene, *α*-pinene and *α*-thujene, were found in fewer than four samples (Tables S1 and S2). Our findings are consistent with earlier reports where medium-chain alkanes (C_13_–C_17_) were found in high percentages in spirulina, albeit with varying relative abundances [23,24,25,27]. The origin of hydrocarbons in cyanobacteria has been attributed to the metabolism of fatty acids through two distinct biosynthetic pathways. One pathway involves a two-step conversion of fatty acids, first to fatty aldehydes, and then to alkanes (or alkenes if the fatty acid contains double bonds). The second pathway proceeds through a mechanism of fatty acid elongation/decarboxylation [42,43,44,45].

**Ketones**: Twenty ketones were identified in this study, with all samples exhibiting a similar number of compounds (Tables S1 and S2). The major contributors to this chemical group were ionone derivatives, including *α*-ionone, *β*-ionone, *β*-ionone epoxide, and dehydro-*β*-ionone. These derivatives were present in all samples, except for dehydro-*β*-ionone, which was detected in 12 samples (Table 3). Notably, *β*-Ionone and *β*-ionone epoxide were the most abundant, with relative abundances ranging from 7.0% to 21.1% and 3.3% to 12.1%, respectively. The presence of *α* and *β*-ionones in spirulina biomass has been reported in previous studies [17,18,23], while *β*-ionone epoxide has been reported in trace amounts in raw spirulina samples [25]. Ionones are produced through the oxidative cleavage of carotenoids by carotene oxygenases [46] and are naturally found in foods containing *β*-carotene [47,48]. Cyanobacteria were previously characterized as a rich source of carotenoids, and the results of this study are consistent with the presence of ionone derivatives in high abundances [49]. Ionones are responsible for the characteristic flowery violet and woody odor. In particular, *β*-ionone contributes to the floral and seaweed odor, *α*-ionone to the violet woody odor, while *β*-ionone epoxide provides a fruity odor [50,51]. Branched ketones such as 6-methylhept-5-en-2-one (sulcatone), 6-methylheptan-2-one, and 2,2,6-trimethylcyclohexan-1-one, previously identified in spirulina biomass [22,23,26], were found in almost all samples at lower relative abundances. 2,2,6-Trimethylcyclohexan-1-one ranging from 0.3% to 2.5% has been reported as the most characteristic ketone in cyanobacteria [49]. This ketone was identified in a previous study [52] as a major volatile component in an eutrophic shallow lake, and its presence was linked with the occurrence of cyanobacteria and algae. Hockelmann [53] also confirmed the formation of this volatile, together with 6-methylhept-5-en-2-one by cultured *Calothrix* sp. and *Plectonema* sp. Furthermore, the nor-carotenoid ketone 6,10-dimethylundeca-5,9-dien-2-one (geranylacetone) was identified for the first time in spirulina food supplements (1.0% to 2.4%). This compound, known for its floral–fruity odor, has been reported as a major volatile product in cyanobacteria [54,55] and algae [56]. Additionally, 3,5,5-Trimethylcyclohex-2-en-1-one (isophorone) was detected in eleven samples (0.4–1.7%) (Table 3) and its presence in spirulina samples has also been confirmed in previous studies [22,26]. In our study, heptan-2-one and octan-2-one were detected in a low relative abundance (0.1–0.8% and 0.02–0.4%, respectively) in thirteen and twelve food supplements, respectively, and they have been previously reported in raw spirulina biomass samples [26] and treated commercial spirulina products [17]. 6,10,14-Trimethylpentadecan-2-one was detected for the first time in spirulina supplements (0.1–1.2%) in twelve samples. This ketone has already been identified in cyanobacteria [57] and green algae [58] and seems to be formed from phytol, a derivative of chlorophyll [59]. Considering that spirulina species are rich in polyunsaturated fatty acids [60], many of the identified ketones can be related to fatty acid oxidation, as enzymes of the lipoxygenase pathway cleave fatty acids to produce these compounds through a sequence of additional reactions [29].

**Aldehydes**: In our analysis of spirulina food supplements, we identified thirty aldehydes, with an average relative abundance of 10.9% across all samples. Seventeen of these volatiles exhibited a cyclic structure and four of them (*β*-cyclocitral, safranal, benzaldehyde and 3,4-dimethylbenzaldehyde) were consistently present in nearly all the samples (Table 3). *β*-Cyclocitral and safranal were the most abundant cyclic aldehydes ranging from 1.2% to 4.2% and 0.6% to 2.4%, respectively. These results align with existing literature data, as these compounds, along with benzaldehyde, have also been found in spirulina samples in high abundances [13,17,22,23,25,26,27]. In contrast, acyclic aldehydes were less prevalent (13 compounds), with hexanal as the primary compound in all samples (1.2% to 8.7%). Previous studies have confirmed the presence of hexanal in spirulina [17,26] and other microalgae [29,61]. Several short-chain aldehydes, including heptanal, oct-2-enal, hept-2-enal, octanal, and nonanal, were detected in more than seven samples (Table 3, Tables S1 and S2). Notably, the last three compounds have not been reported in spirulina biomass. Aldehydes are derived from the chemical and enzymatic oxidation of lipids [29]. *β*-Cyclocitral is formed through the enzymatic degradation of carotenoids [51]. Due to their relatively low odor thresholds, aldehydes can contribute to the distinctive odor of spirulina [26,38]. Hexanal, characterized by a grassy odor and a low odor threshold concentration (OTC) (in water 2.4 μg/L) [62], is easily noticeable in complex matrices [63]. However, in many cases, hexanal is accompanied by unsaturated C6 aldehydes, contributing much more to the overall odor due to their even lower OTCs [62]. *β*-Cyclocitral, with a low OTC value of 3 μg/L [64], imparts a fresh aroma [51]. Lastly, benzaldehyde, known for its characteristic bitter almond-like odor, was confirmed in sixteen spirulina samples (Table 3). This compound is commonly found in various microalgae species and is formed by the enzymatic and chemical degradation of phenylalanine [29].

**Alcohols**: Spirulina food supplements exhibited a substantial presence of alcohols, with an average relative abundance of 8.4% (Figure 3). A notable diversity of alcohols, primarily acyclic, was observed in many samples, with individual compounds ranging from two to sixteen per sample (Tables S1 and S2). Hexan-1-ol, oct-1-en-3-ol and oct-2-en-1-ol were found in almost all samples. Particularly, oct-1-en-3-ol was the most abundant alcohol (2.2 to 8.6%) followed by hexan-1-ol (0.1 to 4.3%) and oct-2-en-1-ol (0.2 to 2.0%). These results are in line with previous investigations of commercial spirulina products and raw spirulina biomass [17,22,26,27], except oct-2-en-1-ol, which had not been previously detected in spirulina samples. Linalool, an alcohol known for its citrus-floral aroma [65], was found in nine samples, consistent with its reported presence in spirulina biomass [25]. Additionally, this study identified other alcohols, such as heptan-1-ol, nonan-1-ol, tetradec-9-en-1-ol and 4,4-dimethylcyclohex-2-en-1-ol, in low abundance (>1%) in more than eight samples (Table 3), and these were not previously reported in spirulina food supplements. The origin of acyclic alcohols in microalgae is attributed to the cleavage of fatty acid hydroperoxides. At the same time, branched compounds can be derived from carbohydrates via glycolysis, or from amino acids via the Ehrlich route [65,66]. Hexan-1-ol is characterized by a herbal odor [17], while C8 alcohols, like oct-1-en-3-ol, contribute a mushroom-like odor, and oct-2-en-1-ol a soapy odor [38]. Since the odor threshold concentrations of alcohols are significantly higher than those of the corresponding aldehydes, they seem to impact the flavor profile of spirulina products less.

**Pyrazines**: Ten alkylpyrazines were detected, each present in abundances below 1% in most cases. Among them, 2,5-Dimethylpyrazine, 2,3,5-trimethylpyrazine and 2,3,5,6-tetramethylpyrazine were found in three samples (Table S1), corroborating previous reports on their presence in spirulina biomass [17,22,26,27], while new compounds in this study were 2,6-diethylpyrazine and 2,5-dimethyl-3-(3-methylbutyl)pyrazine (Table 3). Olfactory studies by Masuda [67] emphasized that monosubstituted pyrazines and substituted methylpyrazines contribute nutty and/or roasted notes, while other alkylpyrazines, excluding decylpyrazine, exhibit a green odor, and higher alkyl-substituted pyrazines possess fatty or waxy odors. The lower alkyl-substituted pyrazines have low odor threshold concentrations and more nutty odors [68]. For example, 2,5-dimethylpyrazine has a nutty odor [38] and might intensify the off-flavor of spirulina [17,22]. Pyrazines are formed during the heat treatment of foods through the Maillard reaction and also via fermentation processes from reactions of amino acids and sugars [69]. However, the spirulina samples in this study were obtained in dry form without specific drying treatment information, and volatile extraction through HS-SPME occurred at a relatively low temperature (60 °C). Further data are required to conclude the pathways for forming these compounds.

**Furans–furanones**: Two furans and two furanones were found in this study (Table 3 and Table S2). Among them, 2-pentylfuran was found in low abundance (≤1.3%) in fourteen samples, consistent with its previous detection in spirulina biomass in several studies [15,17,22,25,26]. This compound originates from the autoxidation of linoleic acid [70] and its flavor threshold in oil at room temperature is 1 mg/L [71]. At elevated concentrations (1–10 mg/L), it exhibits a licorice-like odor with a characteristic beany scent [71]. Conversely, 1-(2,5-dimethylfuran-3-yl)ethanone was present in all samples and has been reported to have a slightly roasted nut-like odor [72]. Its presence in spirulina samples has not been previously reported in the literature. 4,4,7*a*-Trimethyl-6 and 7-dihydro-5*H*-1-benzofuran-2-one (dihydroactinidiolide) were detected in all spirulina supplements in abundances ranging from 1.4 to 4.3%. Dihydroactinidiolide has also been found in previous studies in spirulina samples [13,15,28,73] and in other cyanobacteria such as *Synechococcus* sp. [74]. It has a hay-like [75], pungent violet-like [74] odor with an allelopathic activity that serves as a growth inhibitor for other macrophytes [73,74,76]. Its origin is attributed to the photosensitized oxidation of *β*-ionone [75,77], suggesting that its high abundance is associated with the high *β*-ionone content of spirulina samples (7.0% to 21.1%). 2-Pentylfuran and 1-(2,5-dimethylfuran-3-yl)ethanone are used as flavoring agents by the food industry and their use would not pose a safety concern in the current estimated dietary exposures [78]. However, a very recent toxicity study on 1-(2,5-dimethylfuran-3-yl)ethanone using gpt delta rats indicated that this compound might affect the nasal cavity, liver and lipid metabolism while showing genotoxicity and possible carcinogenicity in the liver [79]. Therefore, a safety re-evaluation for this flavoring agent is necessary.

**Esters and other compounds**: Esters were found in all tested samples showing a low relative abundance compared to other chemical classes of VOCs. In particular, ten individual compounds were identified across various samples constituting on average 1.1% of identified VOCs. Methyl hexadecanoate (methyl palmitate) was detected in fifteen samples and exhibited the highest abundance of all esters ranging from 0.3 to 2.5%. This finding aligns with prior studies, which reported the presence of methyl palmitate in dry spirulina biomass or post-biomass processing [13,24,25,80], along with other ester derivatives in the same chemical class [17,22,26]. Methyl hexanoate, ethenyl hexanoate, and methyl (11*Z*)-hexadec-11-enoate were found in very low abundances in six, seven, and four samples, respectively (Table S1). The presence of diverse ester derivatives has been noted in several cyanobacteria (*Leptolyngbya* sp., *Oscillatoria* sp., *Nostoc* sp.) [81,82], attributed to the reaction of fatty acids and alcohols [80]. Methyl esters of hexadecanoic acid are characterized by a strong oily, fatty, waxy odor [13,81]. Additionally, nitrogenous organic compounds, such as alkyl pyridines and di-*n*-octyl ether were prevalent in all samples (Table 3). Overall, ethers are sparsely identified in spirulina biomass. Specific examples include the detection of benzyl isopentyl ether in previous spirulina studies [22], as well as studies with other microalgae, such as butyl ether in *Chlorella* sp. and *Synechococcus* sp. [26] and octadecyl vinyl ether in *Chlorella* sp. [15].

### 3.3. Variation in Major Volatiles among Spirulina Supplements

**Figure 5:**
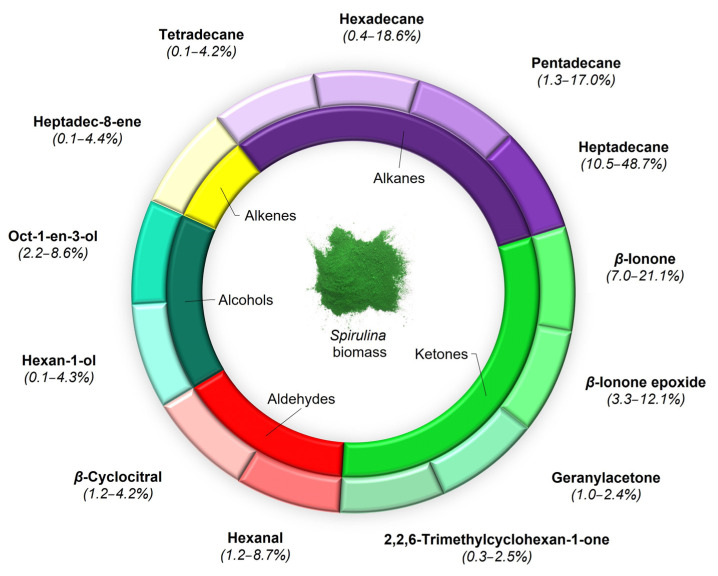
Chemical groups and the most abundant volatiles detected in spirulina supplements. The variation in the relative abundance (%) between samples of each compound is shown in brackets.

Among all VOCs detected in this study, thirteen compounds exhibited the highest abundance, as depicted in Figure 5.

**Figure 6:**
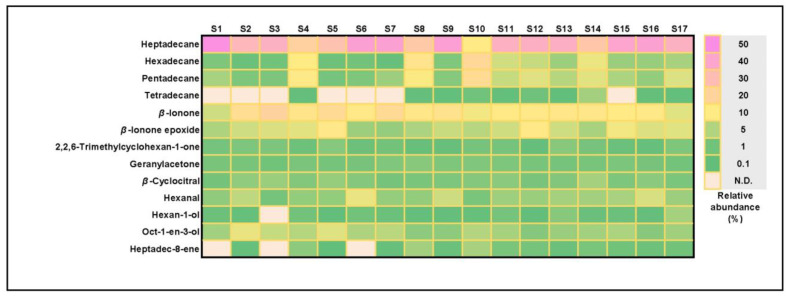
Heatmap of the most abundant volatile compounds showing their distribution and variation in spirulina supplements. For details about the samples, see Table 1. N.D.: Not detected.

Heptadecane, identified in all samples, emerged as the most abundant VOC in this study. Hexadecane, pentadecane, and tetradecane, although showing lower abundance than heptadecane, were still among the most abundant alkanes identified. Hexadecane displayed the highest variation between samples (about 45 times higher value in sample S10 compared to the lowest value detected in sample S7). In comparison, heptadecane demonstrated the lowest variation (4.6 times higher value in sample S1 compared to sample S10). Previous studies have reported heptadecane as a major component in spirulina from Serbia (73.1–82%) [23], Morocco (41.7%) [25], Turkey (39.7%) [24], and Cuba (19%) [27]. The variation of their relative abundance among different samples is visualized in Figure 6.

Ketones, particularly *β*-ionone and *β*-ionone epoxide, consistently stood out as major compounds, aligning with previous findings [17,18,23,25]. These ketones exhibited minimal variation in relative abundance, with a maximum threefold difference among the tested samples (Figure 5 and Figure 6). Additionally, 2,2,6-trimethylcyclohexan-1-one exhibited lower abundances than ionones, followed by 6,10-dimethylundeca-5,9-dien-2-one (geranylacetone), which was identified for the first time in spirulina dietary supplements. Both compounds exhibited slight variation between samples, with samples S13 and S8 showing the highest abundances, respectively (Figure 6). Within aldehydes and alcohols, hexan-1-ol demonstrated significant variability, reaching a 60-fold difference between samples S17 and S5. At the same time, *β*-cyclocitral remains relatively stable, with only S14 displaying a notably higher abundance. These variations of each VOC abundance are likely linked to the substrate and may reflect the biochemical composition of spirulina biomass influenced by external environmental stressors such as temperature, light, pH, or nutrient availability [83]. Considering that the most abundant compounds possess distinct olfactory notes and relatively low sensory thresholds (Table 3), differences in their abundance likely contribute to variations in the sensory quality of spirulina samples.

In a broader sense, the olfactory characteristics of biomass are probably influenced by ketones, aldehydes, alcohols, pyrazines, and furans. Nevertheless, the combination of various odorants ultimately determines the overall olfactory perception of biomass. Further studies are necessary to identify the key odorants in spirulina biomass and elucidate the relationship between the occurring compounds, their abundances, and the perceived sensory quality.

## 4. Conclusions

This study reveals the extensive diversity of VOCs in commercial spirulina food supplements from various geographic regions and manufacturers. This study highlights the potential of spirulina as a reservoir of complex volatile and odorous compounds, potentially contributing to its overall aroma and flavor profiles. A comprehensive workflow was employed for VOC analysis, incorporating HS-SPME extraction, GC-MS analysis, and data processing through AMDIS and the NIST spectral library. In total, 128 VOCs were identified in spirulina food supplement samples, representing various chemical classes such as alkanes, alkenes, ketones, aldehydes, alcohols, esters, pyrazines, and furans. Notably, twelve previously unreported volatile compounds were detected, expanding our knowledge of the chemical composition of spirulina and its potential applications. Key VOCs include dominant hydrocarbons like heptadecane and heptadec-8-ene; carbonylic compounds such as *β*-ionone, 2,2,6-trimethylcyclohexan-1-one; hexanal and alcohols like oct-1-en-3-ol. Many of these compounds are likely contributors to the characteristic sensory qualities of spirulina supplements. Variability in the abundance of VOCs among samples appears to be primarily influenced by the spirulina species, suggesting that the biochemical composition of the spirulina biomass is related to the environmental conditions during growth and processing. The results underscore the potential of spirulina, not only as a dietary supplement, but also as a valuable source of VOCs for extracting beneficial compounds with potential applications in the food, pharmaceutical, and cosmetics industries. Further investigations are needed to explore the associations between volatile and sensory profiles of spirulina and to determine whether VOCs can impact the sensory properties of innovative products, incorporating spirulina biomass as an ingredient. Additional research to identify key odorants in spirulina biomass will facilitate the optimization of spirulina-based food products in terms of their sensory quality.

## Figures and Tables

**Table 1:** Label information on spirulina food supplements used in this study.

Sample	Product Name/Content According to the Label	Origin	Type	Country of Distributor/Producer
S1	Bio-*Spirulina*	Greece	tablets	Greece
S2	*Arthrospira monospira* & *Arthrospira compere*	Greece	flakes	Greece
S3	*Spirulina*	n/a ^1^	tablets	USA
S4	*Spirulina*	n/a	tablets	USA
S5	*Spirulina*	n/a	tablets	USA
S6	*Arthrospira platensis*	n/a	tablets	USA
S7	*Spirulina platensis*	n/a	tablets	Hungary
S8	*Spirulina platensis*	n/a	tablets	England
S9	*Spirulina platensis*	n/a	tablets	Canada
S10	*Spirulina platensis*	Greece	tablets	Greece
S11	*Spirulina platensis*—*Arthrospira platensis*	Netherlands	tablets	Netherlands
S12	*Spirulina*	n/a	capsules	Greece
S13	Bio-*Spirulina*	Greece	tablets	Greece
S14	*Spirulina platensis*	n/a	tablets	Cyprus
S15	*Spirulina platensis*	n/a	capsules	France
S16	*Spirulina platensis*	n/a	powder	n/a
S17	Bio-Spirulina	China	powder	China

^1^ n/a: Not available.

**Table 2:** Identification levels of volatile organic compounds using AMDIS software and related parameters.

Level of Identification	Identification Parameters
A	Retention index ^1^ NET value > 80 using columns A and B Retention time ^2^
B	Retention index ^1^ NET value > 80 using column A Retention time ^2^
C	NET value > 80 using column B Retention time ^2^
D	Retention index ^1^ NET value > 80 using columns A and B
E	Retention index ^1^ NET value > 80 using column A
F	NET value > 80 using column B (peak annotation)

^1^: RI(lib) data available from the NIST MS library; The difference (RI-RI(lib)) was calculated and taken into account in the NET value. ^2^: Verification of the retention time (t_R_) with an authentic standard.

**Table 3:** Volatile compounds detected most frequently (*n* ≥ 4) in spirulina supplements.

A/A	Chemical Compound	Molecular Formula	Level of Identification ^1^	t_R_ (min) Column A/Column B	Number of Samples (*n*)	Relative Abundance (%)	Odor Descriptor
	**Alkanes**						
1	Tridecane	C_13_H_28_	D	8.58/31.62	4	0.05–0.5	alkane ^2^
2	Tetradecane	C_14_H_30_	B	9.49/n.d.	10	0.1–4.2	alkane ^2^
3	Pentadecane	C_15_H_32_	A	10.31/33.42	17	1.3–17.0	alkane ^2^
4	Hexadecane	C_16_H_34_	B	11.12/n.d.	17	0.4–18.6	alkane ^2^
5	Heptadecane	C_17_H_36_	A	11.90/37.80	17	10.5–48.7	alkane ^2^
	**Alkenes**						
6	Hexadec-7-ene	C_16_H_32_	Ε	11.01/n.d.	4	0.03–0.4	n/a
7	Heptadec-8-ene	C_17_H_34_	Ε	11.73/n.d.	14	0.1–4.4	n/a
	**Ketones**						
8	Diphenyl-methanone (benzophenone)	C_3_H_10_O	Ε	11.49/n.d.	5	0.03–1.6	n/a
9	Heptan-2-one	C_7_H_14_O	A	4.26/21.92	13	0.1–0.8	fruity, soapy ^3^
10	6-Methylhept-5-en-2-one (sulcatone)	C_8_H_14_O	D	5.28/24.26	17	0.2–1.1	n/a
11	Octan-2-one	C_8_H_16_O	E	5.33/n.d.	12	0.02–0.4	fruity, soapy ^3^
12	6-Methylheptan-2-one	C_8_H_16_O	D	4.94/23.54	16	0.06–0.5	fruity, sour ^3^
13	3,5,5-Trimethylcyclohex-2-en-1-one (isophorone)	C_9_H_14_O	D	6.16/26.33	11	0.4–1.7	n/a
14	3,4,4-Trimethylcyclohex-2-en-1-one	C_9_H_14_O	D	6.41/26.91	11	0.02–0.3	n/a
15	2,2,6-Trimethylcyclohexan-1-one	C_9_H_16_O	D	5.89/25.77	17	0.3–2.5	n/a
16	5-methyl-3-propan-2-ylidenehex-4-en-2-one	C_10_H_16_O	F	n.d./28.36	13		n/a
17	4-(2,6,6-Trimethylcyclohex-1-yl)-butan-2-one		E	9.86/n.d.	11	0.04–0.2	n/a
18	6,10-Dimethylundeca-5,9-dien-2-one (geranylacetone) *	C_13_H_22_O	D	9.87/33.65	17	1.0–2.4	n/a
19	6,10,14-Trimethylpentadecan-2-one *	C_18_H_36_O	E	12.92/n.d.	12	0.1–1.2	n/a
20	4-(2,6,6-Trimethylcyclohexa-1,3-dien-1-yl)-but-3-en-2-one (dehydro-*β*-ionone)	C_13_H_28_O	E	10.18/n.d.	12	0.3–1.5	violet-like ^3^
21	4-(2,6,6-Trimethyl-1-cyclohexen-1-yl)-3-buten-2-one, (*β*-ionone)	C_13_H_20_O	A	10.21/35.02	17	7.0–21.1	floral ^3^
22	4-(2,2,6-Trimethyl-7-oxabicyclo [4.1.0]heptan-1-yl)-but-3-en-2-one, (*β*-ionone epoxide)	C_13_H_20_O	D	10.24/35.41	17	3.3–12.1	fruity ^3^
23	4-(2,6,6-trimethylcyclohex-2-en-1-yl)but-3-en-2-one (*α*-ionone)	C_13_H_20_O	B	9.72/n.d.	17	0.6–1.7	woody, violet ^2^
	**Aldehydes**						
24	Pentanal	C_5_H_10_O	D	2.46/16.36	13	0.02–0.2	green, fatty ^3^
25	Hexanal	C_6_H_12_O	A	3.28/19.41	17	1.2–8.7	green, grassy ^3^
26	Benzaldehyde	C_7_H_6_O	A	5.08/24.36	16	0.02–1.1	bitter ^3^ almond-like ^3^
27	2-Methylene-hexanal	C_7_H_12_O	E	3.99/n.d.	6	0.02–0.06	n/a
28	Hept-2-enal *	C_7_H_12_O	D	4.98/23.93	16	0.07–1.6	green apple-like ^3^
29	Cyclohexanecarbaldehyde	C_7_H_12_O	F	n.d./23.92	12		n/a
30	Heptanal	C_7_H_14_O	D	4.36/22.16	17	0.1–1.6	citrus-like ^3^
31	Oct-2-enal	C_8_H_14_O	D	6.10/26.25	16	0.2–1.2	fatty, nutty ^3^
32	Octanal *	C_8_H_16_O	D	5.54/24.63	9	0.08–0.3	citrus-like, green ^3^
33	3,4-Dimethylbenzaldehyde	C_9_H_10_O	D	7.40/29.10	16	0.05–0.7	n/a
34	3-Ethylbenzaldehyde	C_9_H_10_O	E	7.50/n.d.	10	0.06–0.1	n/a
35	Nonanal *	C_9_H_18_O	Ε	6.63/n.d.	7	0.1–1.4	citrus-like, soapy ^3^
36	2,6,6-Trimethylcyclohexa-1,3-diene-1-carbaldehyde (safranal)	C_10_H_14_O	D	7.65/29.50	17	0.6–2.4	herb, sweet ^2^
37	2,6,6-Trimethylcyclohex-2-ene-1-carbaldehyde (*α*-cyclocitral)	C_10_H_16_O	D	6.86/27.68	12	0.02–0.5	n/a
38	2,6,6-Trimethyl-1-cyclohexene-1-carboxaldehyde, (*β*-cyclocitral)	C_10_H_16_O	A	7.85/29.93	17	1.2–4.2	minty ^2^
39	2-(2,6,6-Trimethyl-1-cyclohexen-1-yl)-acetaldehyde	C_11_H_18_O	E	8.22/n.d.	12	0.06–0.6	n/a
40	2-Butyl-oct-2-enal	C_12_H_22_O	E	9.26/n.d.	9	0.05–0.3	n/a
	**Alcohols**						
41	Pentan-1-ol	C_5_H_12_O	A	2.70/18.65	9	0.01–0.2	fruity, ethereal ^3^
42	Hexan-1-ol	C_6_H_14_O	A	3.99/21.37	16	0.1–4.3	grassy, marzipan-like ^3^
43	Heptan-1-ol *	C_7_H_16_O	A	5.11/23.85	13	0.04–1.5	fruity, soapy ^3^
44	4,4-Dimethylcyclohex-2-en-1-ol *	C_8_H_14_O	E	5.58/n.d.	10	0.1–0.8	n/a
45	Oct-1-en-3-ol	C_8_H_16_O	A	5.22/23.03	17	2.2–8.6	mushroom-like ^3^
46	Oct-2-en-1-ol *	C_8_H_16_O	D	6.18/26.18	17	0.2–2.0	soapy ^3^
47	2,3-Dimethylcyclohexan-1-ol	C_8_H_16_O	F	n.d./27.8	11		n/a
48	Octan-1-ol	C_8_H_18_O	A	6.21/26.09	4	0.2–0.6	soapy, citrus-like ^3^
49	2-Ethylhexan-1-ol	C_8_H_18_O	D	5.98/25.22	10	0.03–1.2	ethereal, fruity ^3^
50	Νonan-1-ol *	C_9_H_20_O	E	7.27/n.d.	9	0.05–0.5	soapy, fruity ^3^
51	3,7-Dimethylocta-1,6-dien-3-ol (linalool)	C_10_H_18_O	D	6.54/26.72	9	0.03–0.6	citrus-like, flowery ^3^
52	2,4-Di-*tert*-butylphenol	C_14_H_22_O	Β	10.38/n.d.	7	0.01–0.09	phenolic-like ^3^
53	Tetradeca-11,13-dien-1-ol	C_14_H_26_O	E	11.89/n.d.	4	0.03–1.3	n/a
54	Tetradec-9-en-1-ol *	C_14_H_28_O	E	11.68/n.d.	9	0.08–1.3	n/a
	**Pyrazines**						
55	2,6-Diethylpyrazine *	C_8_H_12_N_2_	E	6.31/n.d.	10	0.03–1.5	sweet ^3^
56	2,5-Dimethyl-3-(3-methylbutyl) pyrazine	C_11_H_18_N_2_	E	8.6/n.d.	4	0.3–1.9	n/a
	**Furans—Furanones**						
57	1-(2,5-Dimethylfuran-3-yl)ethanone *	C_8_H_10_O_2_	E	5.99/n.d.	17	0.04–1.3	n/a
58	2-Pentylfuran	C_9_H_14_O	D	5.36/23.79	14	0.05–1.3	n/a
59	4,4,7*a*-Trimethyl-6,7-dihydro-5*H*-1-benzofuran-2-one	C_11_H_16_O_2_	E	10.71/n.d.	17	1.4–4.3	n/a
	**Esters**						
60	Ethenyl hexanoate (*n*-caproic acid vinyl ester)	C_6_H_12_O_2_	E	5.26/n.d.	7	0.05–0.6	sweaty ^3^
61	Methyl hexanoate	C_7_H_14_O_2_	E	4.60/n.d.	6	0.01–0.05	n/a
62	Methyl (11*Z*)-hexadec-11-enoate	C_17_H_32_O_2_	E	13.38/n.d.	4	0.08–0.3	n/a
63	Methyl hexadecanoate (methyl palmitate)	C_17_H_34_O_2_	E	13.48/n.d.	15	0.3–2.5	n/a
	**Other**						
64	2-Pentylpyridine	C_10_H_15_N	E	7.54/n.d.	4	0.2–1.1	fatty, tallow-like ^3^
65	2-Methyl-6-pentylpyridine	C_11_H_17_N	E	8.02/n.d.	6	0.02–0.2	n/a
66	1-Octoxyoctane (di-*n*-octyl ether)	C_16_H_34_O	E	11.6/n.d.	17	0.02–0.9	n/a
67	Propylcyclopropane	C_6_H_12_	F	n.d./21.37	5		n/a
68	5,5-dimethyl-4-propan-2-ylidene-1*H*-pyrazole	C_8_H_14_N_2_	F	n.d./26.32	4		n/a

^1^ For different levels of identification, see Table 2. ^2^ According to the work presented in [37]. ^3^ According to the work presented in [38]. * Compounds identified for the first time in spirulina food supplements. n.d.: not determined; n/a: not available; t_R_: retention time; relative abundance (%): percentage of each compound in relation to the total area of the identified VOCs of each sample.

## Data Availability

The original contributions presented in the study are included in the article/Supplementary Materials, further inquiries can be directed to the corresponding author.

## Supplementary Materials

The following supporting information can be downloaded at: https://www.mdpi.com/article/10.3390/foods13081257/s1, Table S1: Peak areas of the volatile compounds identified in seventeen commercial spirulina food supplements using HS-SPME-GC-MS and column A; Table S2: Peak areas of the volatile compounds identified in seventeen commercial spirulina food supplements using HS-SPME-GC-MS and column B.
